# Introducing graphene quantum dots in decomposable wheat starch-gelatin based nano-biofilms

**DOI:** 10.1038/s41598-024-52560-z

**Published:** 2024-01-24

**Authors:** Marzieh Bakeshlouy Afshar, Ahmad Poursattar Marjani, Peyman Gozali Balkanloo

**Affiliations:** Department of Organic Chemistry, Faculty of Chemistry, Urmia University, Urmia, Iran

**Keywords:** Green chemistry, Materials chemistry, Polymer chemistry

## Abstract

This research aims to discover a viable substitute for the common harmful plastic packaging utilized in food products. Citric acid was employed as an accessible and risk-free precursor in synthesizing graphene quantum dots (GQDs). Using the efficient carbonization technique, GQDs were obtained and subsequently transferred to nano-biofilms in varying percentages relative to natural polymers. FT-IR, XRD, FE-SEM, EDX, and AFM analyses were conducted to examine the formation of the nano-biofilms. GQDs demonstrated optimal performance in the disk diffusion method and the 2,2-diphenyl-1-picrylhydrazyl (DPPH) radical approach. Adding GQDs to starch and gelatin composite improved the physical properties of nano-biofilms such as moisture contact, swelling index, and solubility. The transparency of the films was reduced by GQDs, which reduces the transmission of visible light and plays an important role in food protection. The packaging films' weight loss due to decomposition was examined after being buried in soil for 50 days, which relieved the eco-concerns of these packaging films. To evaluate the performance of the films in inhibiting food spoilage, cherries, and cucumbers were packed with a control film and the fabricated film containing 14 wt% of GQD. After 14 days, the modified nano-biofilm was able to maintain the freshness of the samples.

## Introduction

Annually, a significant portion of manufactured food is annihilated because of carelessness in household utilization^1^. This ultimately causes environmental problems and increases corrosive gasses such as greenhouse gases (Food loss and waste on a worldwide scale contribute to 8% of all human-caused greenhouse gas emissions)^2^. Food packaging is typically designed to safeguard food from pollutants, as well as to prevent microbial impurity and loss of smell. Additionally, it is intended to maintain the product’s modality for an extended time on the shelf^3^. The main foundation of prevalent food packaging methods in today’s industries is the use of plastics obtained from petroleum products. However, this type of packaging safeguards the food against changes in the external and internal conditions of the food. Due to human negligence and lack of waste recycling, these petroleum derivatives accumulate in human habitats and cause various diseases^4^.

Today’s industries have a keen interest in plastic due to the durability and resistance of these materials, which can even withstand high temperatures thanks to their strong carbon chains and diverse additives. However, the difficulty in recycling remains a major issue. Throwing away plastic packaging of food items not only releases harmful chemicals into the environment but also creates a source of pollution due to leftover food. Therefore, it is necessary to discover alternate ways to dispose of plastic packaging to reduce the negative impact on the planet^5^. Insufficient food packaging hinders the United Nations' goal of eradicating worldwide hunger^6^ and results in unfortunate social and economic outcomes, as well as the wastage of essential resources like water and soil^7^.

Active food packaging is not only eco-friendly but also safe and protective of food products. Adding active and antimicrobial substances to polymer substrates prevents the creation of food-contaminating microorganisms^8^. While simple food packaging is designed solely to preserve the food, active food packaging goes a step further by interacting with the food through the absorption or distribution of substances. This innovative type of packaging contains active components that can be applied directly to the food or placed within the packaging space, resulting in a longer shelf-life and better maintenance of the food’s status^9^. For many years, the utilization of nanoparticles in active food packaging has been common. For instance, in 2013, Bikiaris et al. have incorporated copper nanofibers in their research work^10^. The best and most efficient option for active food packaging films is natural polymers (polysaccharides, proteins, etc.), which decompose when abandoned in the environment, unlike synthetic polymers^11^. Starch is an excellent biopolymer for active food packaging, due to processing advantages such as availability, low cost, and biodegradability. However, it falls short of synthetic polymers in terms of mechanical properties. Studies have shown that the use of starch is limited due to the poor tensile strength properties of pure starch layers, and gelatin alone has little use due to its poor plasticity, but the integration of these two materials improves the properties of both. By mixing starch and gelatin, the molecular chains of starch are uniformly dispersed in the gelatin network. They are strengthened by hydrogen bonds, and as a result, the tensile strength and breaking of the length of the gelatin layer improves the performance of starch. The possible interactions between starch and gelatin show the C–O–C glycosidic bond that interacts with the gelatin macromolecule. Then, ionized connections are formed, which due to the hygroscopic nature can be related to the water in the starch structure. In the second step, the interaction of hydroxyl groups with hydrogen bonds is shown. Adding glycerin to the polymer matrix increases the number of hydroxyl groups in the system and acts as a cross-linker. Subsequently, new active donor–acceptor sites increase in the polymer matrix. An opportunity for nucleophilic attack on the graphene quantum dots molecule has been created, which can both connect to the structure of the polymer matrix chains in the carbon atom of the carbonyl group and interact with the parts of the glycerin chains through proton exchange or the formation of less durable hydrogen bonds^12^. For instance, it has been documented that incorporating corn starch with gelatin in grape packaging films has improved the mechanical properties of resulting composites such as enhanced solubility and reduced *water vapor permeability* (WVP)^13^. Another study revealed that enriching the quantity of gelatin in the cassava starch/gelatin composite enhanced the pliability and softness of the packaging films, making this composite a practical preference for active food packaging^14^.

The use of gelatin is believed to have environmental benefits as it is produced by recycling meat waste through the hydrolysis of collagen found in animal bodies^15^. Starch is produced through the linkage of polyhydroxy compounds, specifically amylopectin, and amylose molecules, which are connected by hydrogen bonds^16^. Active food packaging serves the crucial purpose of preserving the safety and quality of food by utilizing antimicrobial substances, while also ensuring that the appearance and overall appeal of the food remain unchanged^17^. In the quest for further enhancing the functionality of packaging materials, scientists have turned their attention to the incorporation of GQDs into polymer matrices^18^. GQDs, which are nanoscale carbon structures, possess remarkable antimicrobial properties due to their high surface area and unique chemical composition^19^. When integrated into the polymer matrix of packaging materials, GQDs can effectively inhibit the growth of bacteria, fungi, and other harmful microorganisms, thus extending the shelf life of perishable goods. Furthermore, GQDs exhibit excellent stability, biocompatibility, and low toxicity, making them a viable choice for antimicrobial applications in the food and healthcare industries^20^.

The origin of GQDs dates back to 2008 when Ponomarenko and Geim were able to discover GQDs based on the studies done on carbon dots (CDs)^21^. GQDs, which belong to the carbon quantum dot (CQD) group, possess antimicrobial properties and have a minuscule size of just 2–10 nm^22^. This aligns with the European Commission’s definition of nanoparticles, which stipulates that a substance is considered a nanoparticle if over more than its total digit of particles has an outer measurement between 1 and 100 nm. GQDs exhibit antimicrobial properties by disrupting the bacterial envelop and depleting intracellular components, leading to elevated levels of *reactive oxygen species* (ROS) and subsequent eradication of both gram-positive and gram-negative bacteria. Similarly, implementing GQDs has proven to be effective in boosting durability, lessening the weight, and making the satisfactory texture of packaging films^23^. Their distinctive characteristics, including their proficiency to be decomposable, meager toxicity, and high solubility due to free functional groups, as well as their antioxidant and antifungal properties, have made quantum dots a popular option for active food packaging. For instance, Ezati et al. have demonstrated that films composed of chitosan/gelatin have effectively suppressed the growth of spoilage bacteria *L. monocytogenes* and *Escherichia coli* (*E. coli*) in avocados^24^. Alginate-based films, along with silicon quantum dots have been used in another research^25^. In 2011, the *US Food and Drug Administration* (FDA), during a study, proved that quantum dots are safe in terms of toxicity and reliable for biological purposes^26^.

There are two primary methods for synthesizing GQDs, as well as other quantum dots. The first approach is known as the top-down and involves using physical and chemical ways to dissolve various carbon materials such as graphite, carbon tubes, and candle soot^27^ into smaller nano-sized pieces. The second approach is called the bottom-up and pertains to incorporating small organic molecules through hydrothermal or carbonization techniques. The bottom-up approach has become increasingly popular due to its performance and the use of readily obtainable organic sources such as glucose, citric acid, and saccharides^28^. Unlike the first approach, bottom-up eliminates the requirement for advanced equipment and harsh reaction conditions^29^. Among the bottom-up approach, the thermal method has become widespread because the synthesized quantum dots are not harmful to the ecosystem^30^. The fundamental principle of the thermal technique is to rapidly lengthen the carbon chain of organic materials that are based on carbon without using any solvents^31^. An instance of this is using the pyrolysis of citric acid at 200 °C to obtain eco-friendly GQDs within 20 min^32^, which is the same strategy employed in this present study.

In this research, GQD was acquired through citric acid pyrolysis, a swift and straightforward method. Citric acid is a safe and inherently arising organic acid, including three carboxylic groups, which makes it reasonable for benefit as a food additive^33^. It was then incorporated as an active and antimicrobial agent into films composed of natural biopolymers such as starch and gelatin. The efficiency of GQD as biocompatibility nano-fillers in active packaging polymer substrates was examined through a series of analyses, including *Fourier-transform infrared spectroscopy* (FT-IR), *field emission scanning electron microscopes* (FE-SEM), *X-Ray diffraction analysis* (XRD), *energy dispersive X-ray analysis* (EDX), and *atomic force microscopy* (AFM), as well as evaluation of mechanical, optical, antibacterial, antifungal, and antioxidant properties of the films. Nano-biofilms were applied to cherries and cucumbers to evaluate their effectiveness in safeguarding against nutrient loss and tissue damage, as these perishable fruits and vegetables tend to rapidly loss their vital nutrients and moisture, leading to withering and deterioration. This study is expected to contribute to advancing nanotechnology-based safe food packaging with an organic chemistry approach. By combining the advantages of starch and gelatin-based packaging materials with the antimicrobial properties of GQDs, this study is paving the way for a new generation of sustainable and hygienic packaging solutions. The incorporation of GQDs into starch and gelatin matrices not only enhances the barrier properties and mechanical strength of the packaging but also provides an additional layer of protection against microbial contamination. This innovative approach holds great promise for addressing the pressing challenges associated with food safety, waste reduction, and environmental sustainability, ushering in a future where packaging not only preserves the freshness of products but also contributes to a healthier and greener world.

## Material and methods

### Materials

Wheat starch crystals (MW: 162.14 g/mol, Density: 1.5 g/cm^3^ (20 °C)) were purchased from Spectrum Chemical. Animal gelatin powder (Type A) was purchased from Innovating Science. Glycerin (MW: 92.09 g/mol, Density: 1.26 g/cm^3^ (20 °C)), citric acid monohydrate (MW: 192.13 g/mol, Density: 1.665 g/cm^3^ (18 °C)), and sodium hydroxide pellets (MW: 40 g/mol, Density: 2.13 g/cm^3^ (20 °C)) were obtained from Merck Chemicals Co (Germany) 2,2-diphenyl-1-picrylhydrazyl (DPPH) was obtained from Sigma-Aldrich (USA). All materials are employed without any purification.

### Methods

#### Synthesis of GQD

To synthesize GQD, the same procedure as the Dong et al. method was followed^34^. 6 g citric acid monohydrate was weighed and transferred to a 50 ml beaker. Then, it was heated up to 200 °C using a heater. After roughly 4–5 min of heating, the powdered citric acid modifications phase into a liquid state. During the synthesis process, the color and state of the liquid change, as detailed in Table 1, for a duration of up to 20 min. Finally, to achieve a neutral pH (7.0), 20 mL NaOH (1 M) was added to dark orange liquid, while being continuously stirred strongly. After stirring for half an hour, resulting in the formation of GQD. The viscous liquid was placed in an oven at 120 °C for 40 min to acquire the solid sample of GQDs.

**Table 1:** Ocular changes in citric acid during GQD synthesis.

Time	Minute 0	Minute 5	Minute 10	Minute 15	Minute 20
Color	White	Colorless	Pale yellow	Orange	Dark orange
State	Powder	Liquid	Boiling liquid	Thick liquid	Viscous liquid

#### Preparation of nano-biofilms based on natural polymers

**Figure 1:**
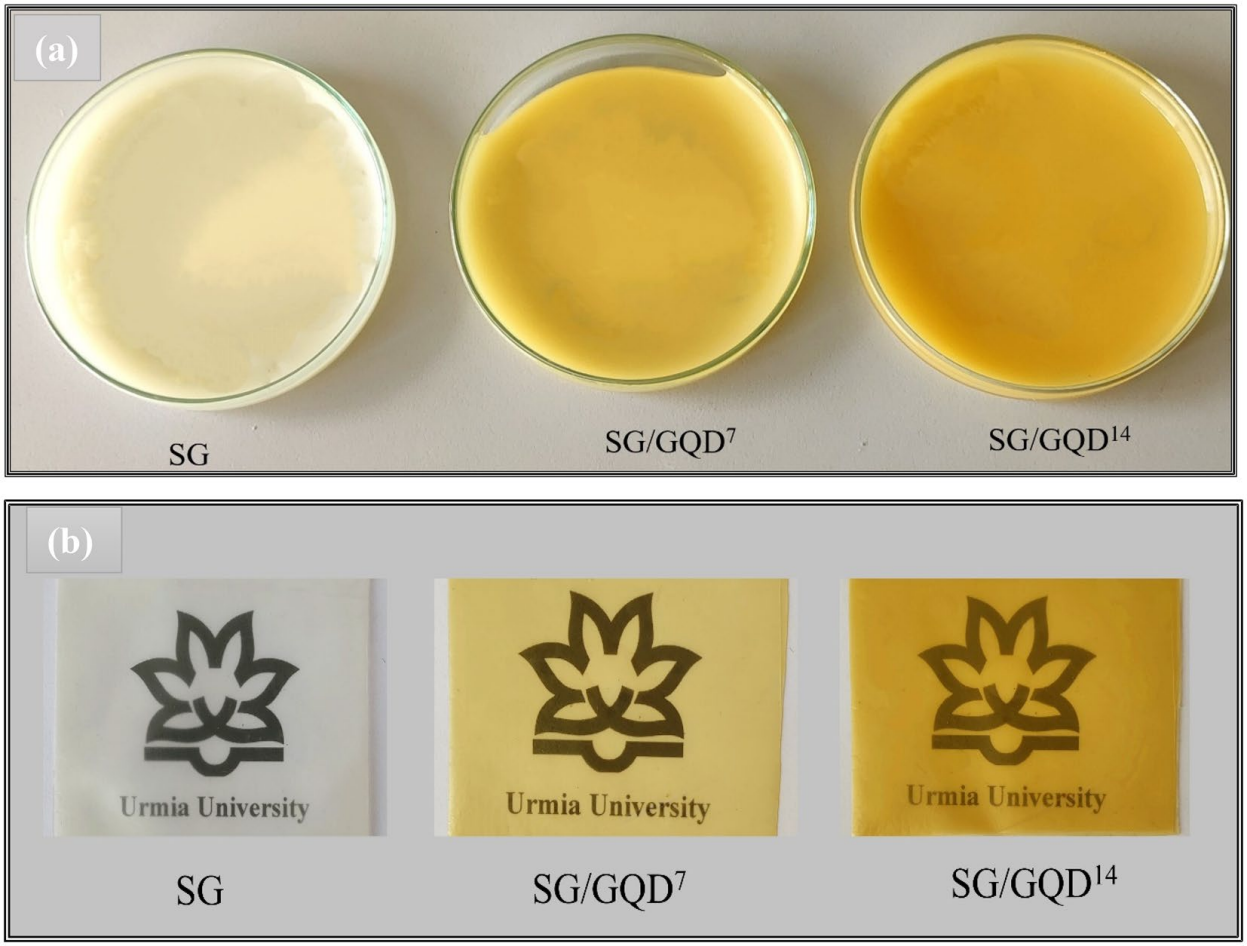
Digital images before (**a**) and after (**b**) drying for starch/gelatin neat biofilm and nano-biofilms containing 7 wt% and 14 wt% of GQD.

Nano-biofilms were prepared by casting technique, which is a conventional method in the fabrication of polymer substrates for active food packaging^8,24,25,35^. In short, 3 g of starch (S) and 1 g of gelatin (G) (75:25) were poured into a beaker with 100 mL of distilled water and heated to 90 °C with a heater stirrer. After an hour, the solution turned into a gel. At the end of gelation, 1.6 g of glycerin (40 wt% based on polymers) was added. Then 7 and 14 wt% of GQD (based on total polymers in dry state) were added to the mixture (SG), and it was vigorously stirred for half an hour. The film’s solution was poured into petri dishes with a diameter of 8 and 2 cm high (to the amount of 10 g), and dried in ambient air for 48 h. Afterward, the films were separated from the container and were recognized as SG/GQD^7^ and SG/GQD^14^. To prepare the control sample film (SG), the previous method was adopted (Fig. 1).

#### Structure of nano-biofilms

To analyze the chemical composition of the films and investigate the influence of GQDs, as well as discover any functional groups present, FT-IR (Bruker IFS-66/S FT-IR) was employed within the 400–4000 cm^−1^ wavenumber range. XRD was used to expose the film samples. As a result, the XRD Bruker D8 Advance determined the crystal structure of the GQDs and how they are dispersed in the polymer substrate. The FE-SEM TESCAN MIRA2 was used to investigate the morphology, composition, and structure of the surface of films at the nanometer scale. For EDX analysis, the TESCAN MIRA2 device was used to detect the type of elements and the weight percentage of each in nano-biofilms. Nanowizard 2 microscope (maximum surface roughness is 5 microns and imaging accuracy is 0.1 nm) was used for AFM analysis to identify GQD nanoparticles on the surface of nano-biofilms and to image the surface topography of the sample.

#### Thickness of nano-biofilms

The thickness of each film specimen was determined by appointing 4–5 parts from films with different GQD percentages, which were completely smooth and monotone. These parts were measured with Insize Digital Outside Micrometer 25–3108 A, and the averaging method was operated to discern the final thickness.

#### Moisture contact (MC)

To determine the MC for the fabricated films, the weight loss method was employed. Initially, portions with dimensions 2 × 2 cm^2^ of the films were cut and weighed. Then, the specimens were dried in an oven at 70 °C ± 2 for a day and weighed similarly^36^. The MC of the films can be calculated using the following formula:

1 $${\text{M}}_{{\text{C}}} = [(W_{w} - W_{d} ) \div W_{w} ] \times 100\user2{\% }$$

where *M*_*C*_ is the moisture contact expressed as a percentage, *W*_*w*_ is the content of the pre-parched sample, and *W*_*d*_ is the content of the parched sample.

#### Swelling index

2 × 2 cm^2^ sizes were cut from the films, dried at 70 °C for 24 h, and weighed (m_1_). These dehumidified samples were then immersed in 30 mL of distilled water for 5 min, excess wet was removed, and weighed (m_2_)^37^. The amount of water absorbed by the sample was evaluated using the following equation:

2 $${\text{SI}}=\frac{\left({m}_{2- }{m}_{1}\right)}{{m}_{1}}\times 100\%$$

where *SI* is swelling index percentage, and *m*_*1*_ and *m*_*2*_ refer to dried samples and swelled samples, respectively.

#### Solubility

To calculate the solubility percentage of polymer films, 2 × 2 cm^2^ sizes of the samples were dried at 70 °C and weighed. Then, the samples were immersed in a beaker containing double distilled water for 24 h. At the end, after being removed from the beaker, the samples were dried in an oven at 70 °C for 1 day and then weighed (m_3_)^38^. The solubility can be calculated using the following equation:

3 $${\text{S}}=\frac{{(m}_{1}-{m}_{3})}{{m}_{1}}\times 100\%$$

where *S* is solubility percentage, *m*_*1*_ and *m*_*3*_ refer to dried samples’ weight (before immersion in double distilled water), and re-dried samples’ weight, respectively.

#### Opacity of SG/GQD nano-biofilms

In order to the opacity of the film, the absorbance of each 3 cm × 1 cm section of the film is gauged at a wavelength of 600 nm, with an empty cell serving as a reference. When placing the sample inside the cell, care was taken not to create any gaps. These absorbance readings are subsequently utilized in a formula to compute the opacity^39^.

4 $${\text{O}}= \frac{{Abs}_{600}}{D(mm)}$$

where *O* is Opacity, *Abs*_*600*_ is the absorbance of samples at a wavelength of 600 nm, and *D* is thickness.

#### Transmittance of SG/GQD nano-biofilms

The UV–Vis spectrophotometer was utilized to discern the transmittance of both the unmodified SG film and the SG/GQD nano-biofilms. Spectral measurements were taken by cutting the film instances into 4 cm^2^ and located in a spectrophotometer cuvette. The target wavelength was 280 and 660 nm^40^.

#### Penetration test of GQDs in food

Acetic acid aqueous solution (3% w/v) was operated as a virtual food model. The hydrated films were sliced into 1 cm^2^ fragments and submerged in a 5 mL virtual nutrient solution. Subsequently, at specific time intervals ranging from 10 to 60 min, the films were extracted, and UV–Vis spectroscopy was recorded from the residual solution^11^.

#### Decomposability of nano-biofilms

To assess the decomposability of the synthesized packaging nano bio-films, 1 cm^2^ pieces of the films were initially cut and weighed. Subsequently, these samples were placed in containers filled with moist garden soil. Over 50 days, the samples were periodically extracted from the soil every 5 days, weighed, and their weight loss percentage was calculated using Eq. (5)^41^:

5 $$\mathrm{Weight \,loss }(\mathrm{\%})=\frac{{W}_{i}- {W}_{f}}{{W}_{i}}\times 100$$

where *W*_*i*_ and *W*_*f*_ are the initial weight and final weight of samples.

#### Mechanical analysis

Three tests, namely tensile strength (TS), elongation at break (EAB), and Young’s modulus (YM) were conducted using a Santam 250 KN tissue analyzer to evaluate the mechanical properties of the films. The machine applied a minimum force of 300 kg, while the film pieces measured 10 nm × 70 nm and were left at a temperature of 23 °C and relative humidity of 50% for 1 day before undergoing testing in the analyzer^42^.

#### The application of nano-biofilms in the protection of real food samples

To evaluate the effectiveness of GQD nano-biofilms in preserving the freshness of perishable fruits and vegetables such as cherries and cucumbers, the antimicrobial performance of nano-biofilms was assessed. Specifically, the antimicrobial impact of the SG/GQD^14^ film was investigated on cherries and cucumbers due to their sensitivity to mold growth. To conduct this evaluation, fresh fruits and vegetables were submerged in film solution for 1–2 min and subsequently air-dried. The real food samples, both coated and uncoated with SG and SG/GQD^14^ solutions, were placed in trays, and stored in a humid environment at a temperature of 25 °C. Daily examinations were conducted over 14 days to observe any visible changes on the sample surfaces. The observations were documented for further analysis. To conduct a more detailed examination, the extent of fruit decay was determined by calculating the weight loss using a formula resembling "Decomposability of nano-biofilms"section. where, this time ${W}_{i}$ is the weight of the fresh fruits packed with films at the beginning of the packing (0 day) and ${W}_{f}$ is the weight of fruits packed after 14 days^40^.

#### Assessment of antioxidant capacity by DPPH radical method

To extract the antioxidant from the film selection, the combination of 50 mg of the sample and 5 mL methanol was set aside for 20 min. In a separate receptacle, 4 mg of DPPH (2,2-diphenyl-1-picrylhydrazyl) and 100 mL of methanol were incorporated and left in the dark for half an hour to acquire the DPPH solution. The resulting solution was then counted for absorbance at 515 nm, and the antioxidant action was calculated using the following formula^43^:

6 $${\text{I}}\%=\left[\left({A}_{blank}-{A}_{sample}\right)/{A}_{blank}\right]\times 100$$

where *I* is the percentage of antioxidation activity, *A*_*blank*_ and *A*_*sample*_ refer to the absorbance of the blank and main sample, respectively.

#### Antimicrobial attributes

The standard disk diffusion technique was applied to determine the antimicrobial efficacy of the packaging films. The foodborne pathogens *E. coli* (ATCC 25,922) and *Listeria monocytogenes* (ATCC 19,115) were assigned as representative microorganisms. Muller Hinton agar was prepared, and the spread plate technique was used to inoculate the pathogens onto the agar plates. The packaging film discs were then placed on the texture of the agar plates and incubated at 37 °C for 24 h. The diameter of the inhibition zone encircling the discs was gauged^44^.

## Result and discussion

### FT-IR analysis

The interaction between the constituents of each film and electromagnetic radiation is reflected in the FT-IR spectrum^45^, which was used to investigate the change in condition and interpretation of the polymer substrates of the films before and after the addition of quantum dots. The analysis was conducted on the control sample and those with varying rates of GQD.

**Figure 2:**
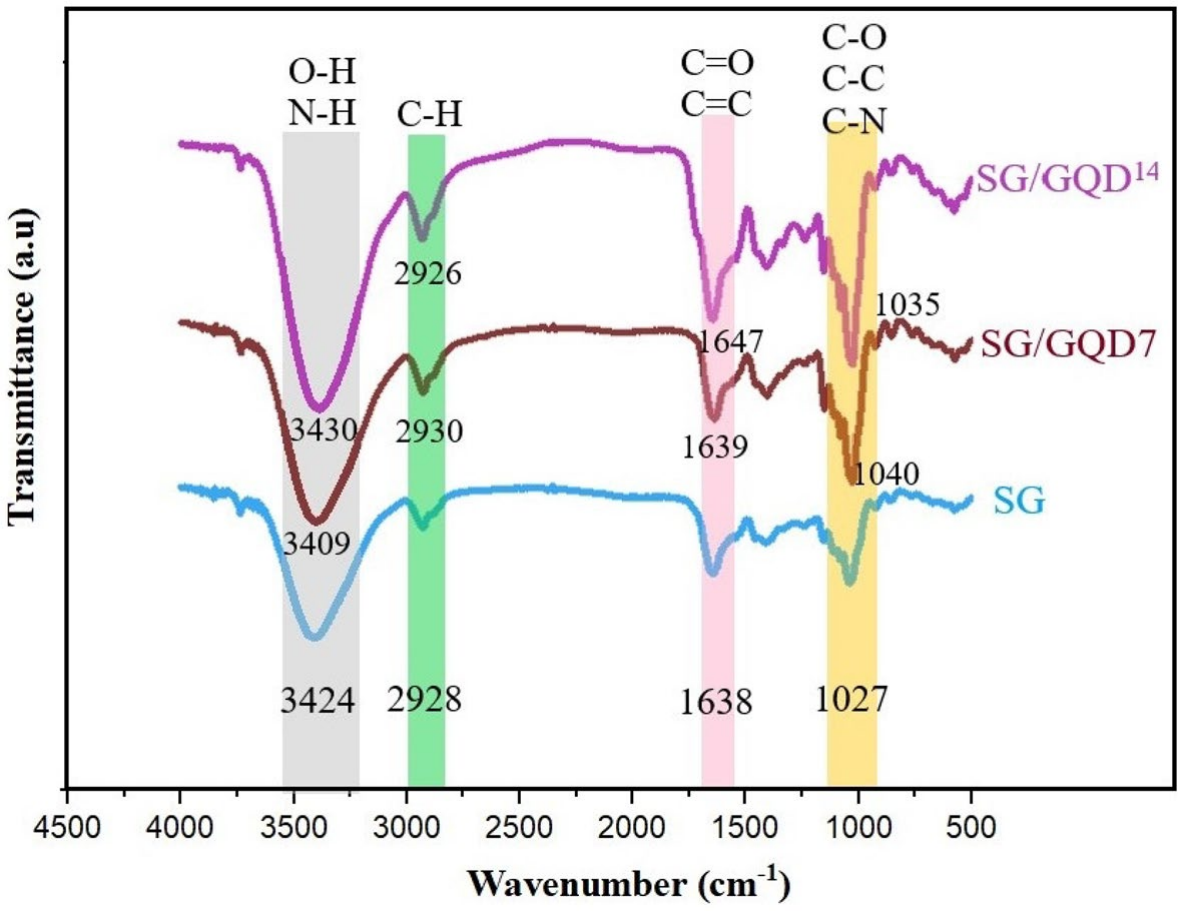
The FT-IR spectra of starch/gelatin neat biofilm and nano-biofilms containing 7 wt% and 14 wt% of GQD.

To identify the organic molecules attending in the samples, a range of 400–4000 cm^−1^ wavenumber was selected for this investigation. The FT-IR spectra for the three types of films (SG, SG/GQD^7^, and SG/GQD^14^) display striking similarities, and each functional group peak shows slight variations (Fig. 2). These outcomes imply that the incorporation of GQD into the biopolymer matrix was done with great care and suggest that the chemical composition of the substrate was not altered. Furthermore, the excellent dispersal of GQD attests to the compatibility between GQD and the polymer substrate, which is largely attributed to the high solubility of quantum dots^8^.

**Figure 3:**
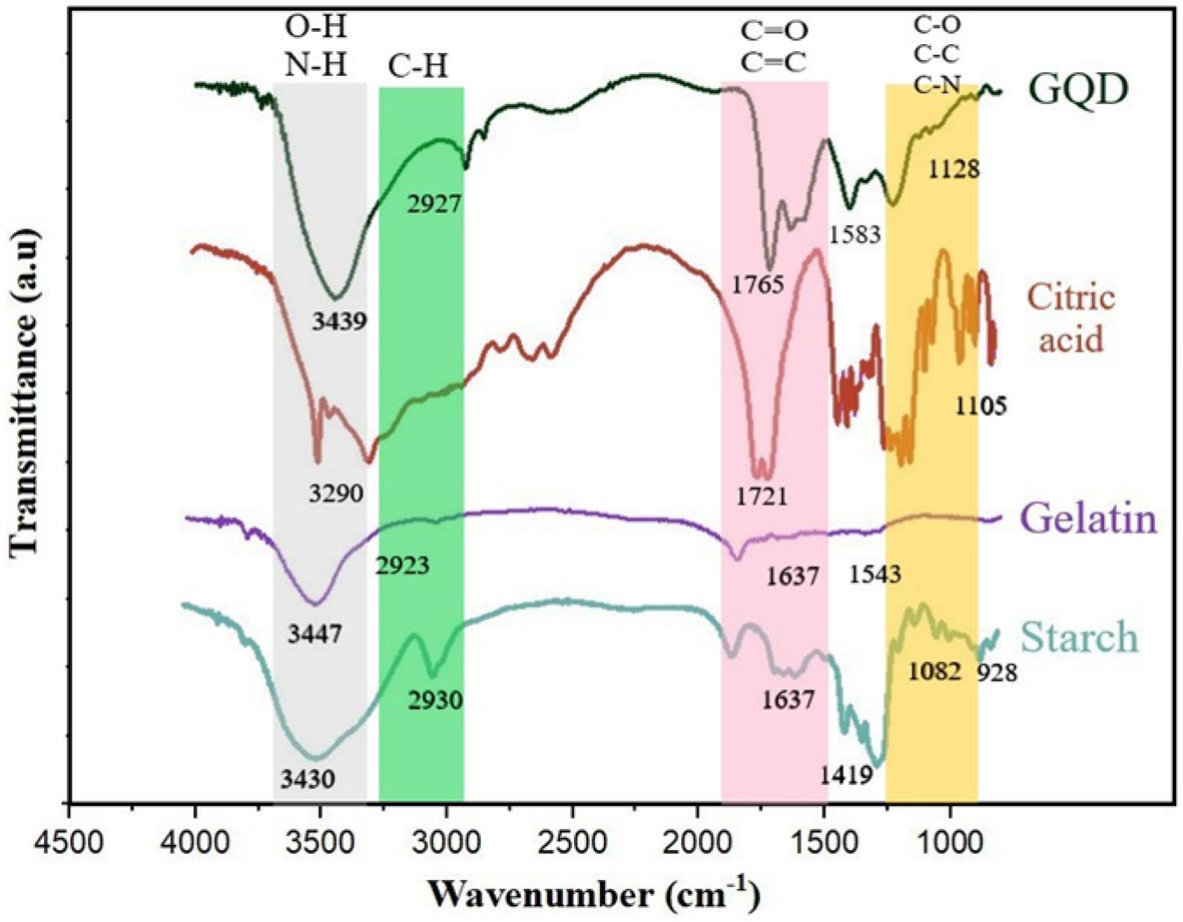
The FTIR spectra of starch/gelatin neat biofilm and nano-biofilms containing 7 wt% and 14 wt% of GQD.

The following, FT-IR spectrum of components, namely starch, gelatin, graphene quantum dots, and citric acid, is presented in Fig. 3. At the starting point of the FT-IR spectrum of starch, there was a clear and index peak at 3430 cm^−1^, which corresponds to O–H stretching is expected to the existence of glucopyranose rings in polysaccharides. 2930 cm^−1^ was the existence of C–CH_2_ in the form of asymmetric stretching vibration of monomer. At 1637 cm^−1^, the peak corresponding to the O–H conformation change was seen in the water molecule entering the starch. The peak at 1419 cm^−1^ was allocated to the bending vibration of C-H in the –CH_2_ and –OH complex. The stretching vibration of the C=O and C–C bond, moreover the bending vibration of C–OH was related to 1082 cm^−1^. There was a weak peak at 928 cm^−1^, indicating an OH–C_6_ bond in starch^46^.

Significant absorption peaks are observed in the FT-IR of gelatin structure due to the presence of amide groups. The stretchy C=O related to CO_2_ in a hydrogen-bonded form (often with water) is apperceived in the amide-A range at 3447 cm^−1^. N–H bending and C–N stretching from amide-B was at 2923 cm^−1^. Amide-1,2 and 3 occurred in 1637, 1543, and 1258 cm^−1^, respectively^47^.

The FT-IR spectrum of citric acid demonstrates definite peaks at 3290 cm^−1^ (OH stretching vibration), 1721 cm^−1^ (C=O stretching vibration), 1105 cm^−1^ (C–OH stretching vibration), and 778 cm^−1^ (CH_2_ shaking)^48^. The GQD spectrum was explored by managing distinct peaks. –OH and CH_2_ stretching vibrations appeared at 3439 and 2927 cm^−1^. The 1765 cm^−1^ belonged to the stretching vibration of C=O. There were bending (NH_2_) and stretching vibrations of the double bond of C=C at 1583 cm^−1^ and C–N at 1128 cm^−1^^34^.

### XRD

XRD analysis can discern the structure of both crystalline and non-crystalline materials by eliciting scattered beams when uncovered to X-rays^49^. This strategy equips worthwhile communication on traits such as middle grain size, grade of crystallinity, and phases. Single crystal selections form point patterns, while polycrystalline selections form ring patterns^50^.

**Figure 4:**
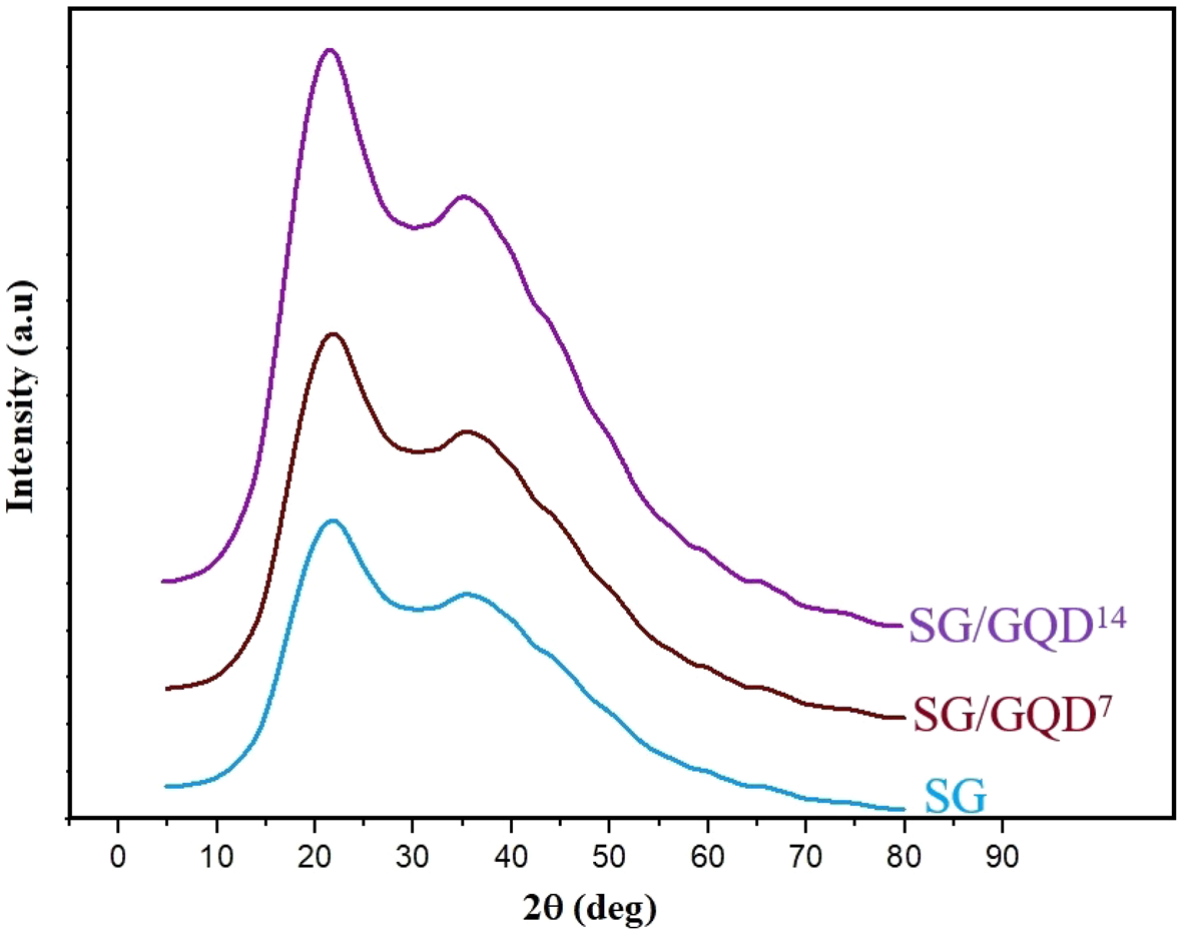
XRD spectra of starch/gelatin neat biofilm and nano-biofilms containing 7 wt% and 14 wt% of GQD.

Figure 4 shows the XRD spectrum of packaging films with varying amounts of GQDs; it can be observed that all three nano-biofilms exhibit a broad diffraction peak at about 2$\theta$ = 21.5°, which is associated with gelatin^24^.

By incorporating GQDs into the polymer substrate (in SG/GQD^7^ and SG/GQD^14^), the peak becomes sharper, and its intensity increases, reaching its peak value in the SG/GQD^14^ film. This is attributed to the appearance and intensification of the peak associated with GQD at a similar degree (2$\theta =$ 22^∘^). GQDs fall under the category of materials with superb crystallinity owing to the graphene networks present in their structure, resulting in a peak at 2$\theta =$ 22^∘^ in their XRD pattern. This pattern closely resembles that of graphite, which can be attributed to the heightened carbonization of citric acid during the synthesis process leading to the formation of GQDs^51^.

The broadness of the existing peaks suggests an amorphous structure of the films. The synthesized GQD exhibits a layer-to-layer distance of approximately 0.39 nm, indicating the layered structure of GQDs. This is consistent with interlayer distance values reported for GQDs synthesized through different methods (0.340 $nm<x<0.481 nm$). The crystal size of GQDs was calculated as 37.1 nm using the Scherrer Eq. ^52^.

### Scrutinizing the morphology, arrangement, and structure of the films

FE-SEM is a cutting-edge imaging method that allows scientists to examine the surface morphology and characteristics of various specimens^53^. FE-SEM achieves this by utilizing a focused electron beam with precise energy and wavelength to scan the sample surface, producing high-resolution images and gathering crucial data on its surface feature. Compared to conventional SEM, FE-SEM boasts superior resolution thanks to its field emission source of electrons. FE-SEM simplifies the depiction and imaging of a significance in its elemental form. Compared to TEM, this method has been verified to be more efficient at lower voltages as well as at fewer spatial resolutions and has made it conceivable to explore among nanoparticles and other small-sized systems^54^.

**Figure 5:**
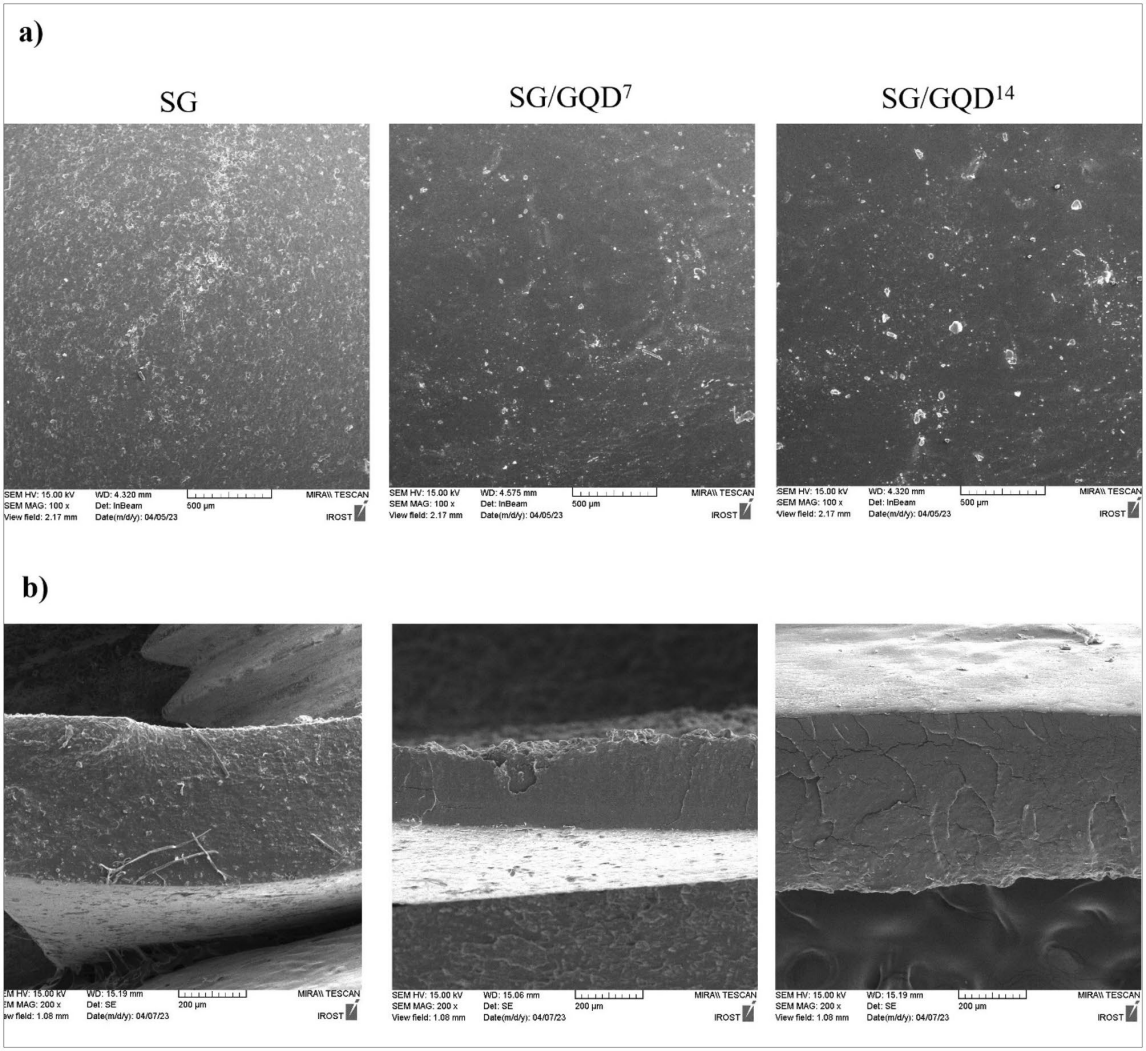
FE-SEM images of starch/gelatin neat biofilm and nano-biofilms containing 7 wt% and 14 wt% of GQD (**a**), surface and cross-sectional (**b**).

**Figure 6:**
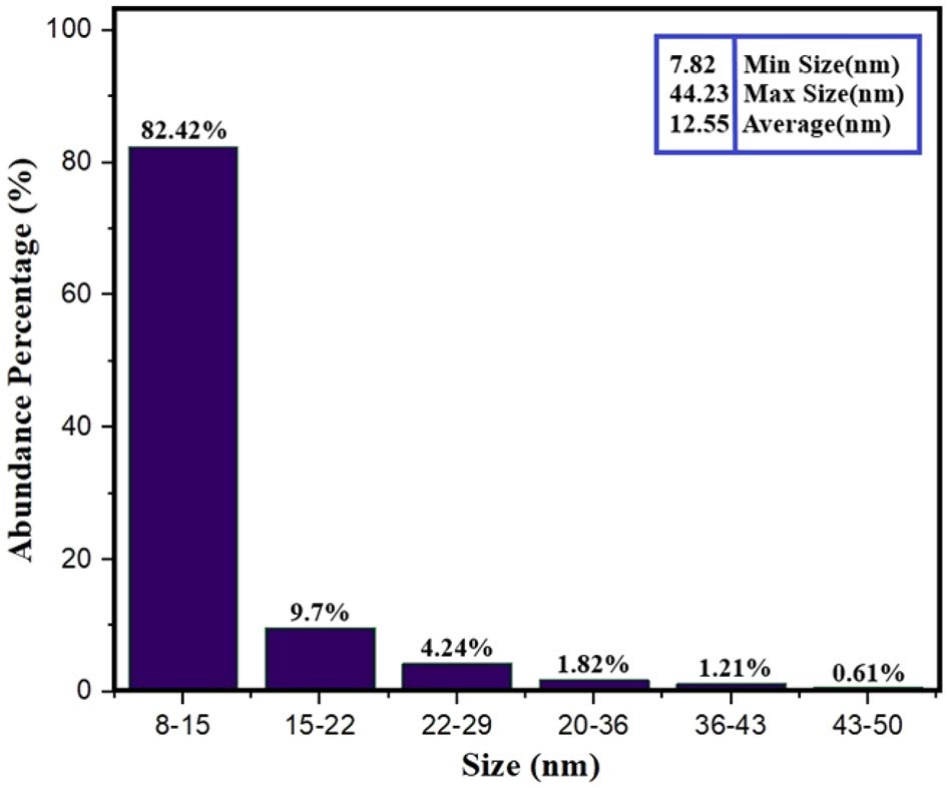
Abundance percentages of particles by FE-SEM.

An analysis was carried out to analyze the arrangement of nano-biofilms (Fig. 5). This involved investigating the consistency and uniformity of the layers, the distribution and behavior of GQD in the biopolymer matrix, and the distribution of GQD as a filler in the substrate using FE-SEM. The control film showed a satisfactory texture without any damages or flaws, although some empty holes were observed. GQD, possessing hydrophilic and polar functional groups at the edges and a high surface-to-volume ratio^55^, seamlessly integrated with the matrix and occupied the voids. Still, Javanbakht et al. noted that GQD led to accumulations in certain areas of the film^56^, slightly enhancing its surface roughness due to nanoparticles proliferation. Despite this, GQD’s nano-filler properties make it an invaluable addition as it enhances film permeability and provides a robust guard against external factors such as microbial spoilage. The average particle size distribution was measured from 8 to 50 nm due to different aggregations, and the percent abundance of each is given in Fig. 6. The most abundant percentage is related to the size of 8–15 nm, which is 82.42%, so the average size of the particles is estimated to be 12.5 nm.

EDX analysis is an add-on to FE-SEM microscopy that allows for determining element percentages in samples. By utilizing the unique X-ray energy emitted from a nano-biofilm sample containing GQDs, this analysis can identify the type of element present and its weight or atomic percentages.

**Figure 7:**
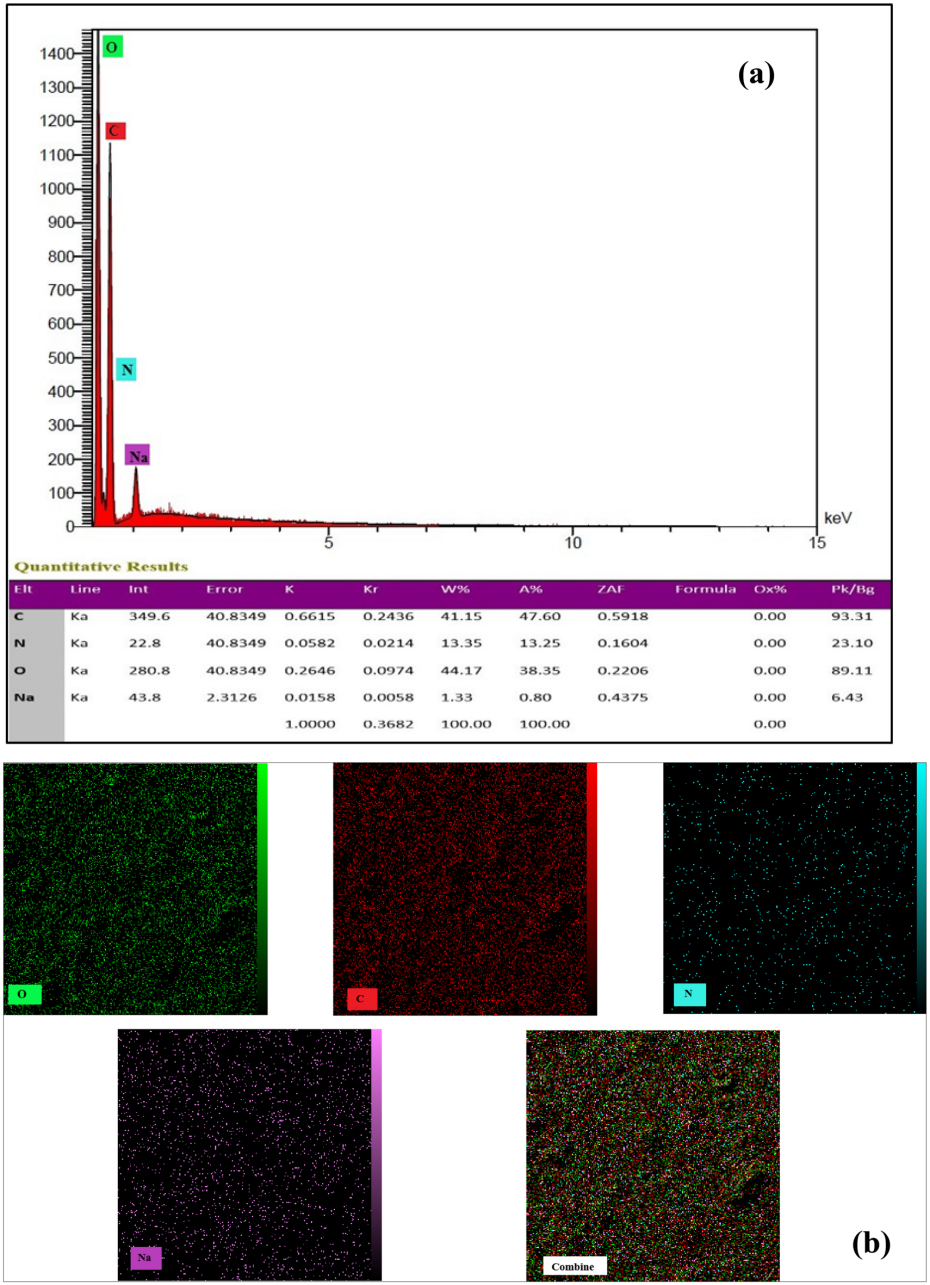
EDX (**a**) and elemental mapping (**b**) of nano-biofilms.

In Fig. 7a, the EDX diagram illustrates the X-ray energy diffraction of a nano-biofilm containing 14% graphene quantum dot (SG/GQD^14^). The identified elements in the sample include oxygen (44.17%), carbon (41.15%), nitrogen (13.35%), and sodium (1.33%). Higher intensity signals of oxygen and carbon elements were observed.

To measure the homogeneity of the nano-biofilms, elemental mapping was conducted, and as indicated in Fig. 7b, the even dispersion of O, C, N, and Na elements within the film serves as confirmation.

### AFM analysis of nano-biofilms

**Figure 8:**
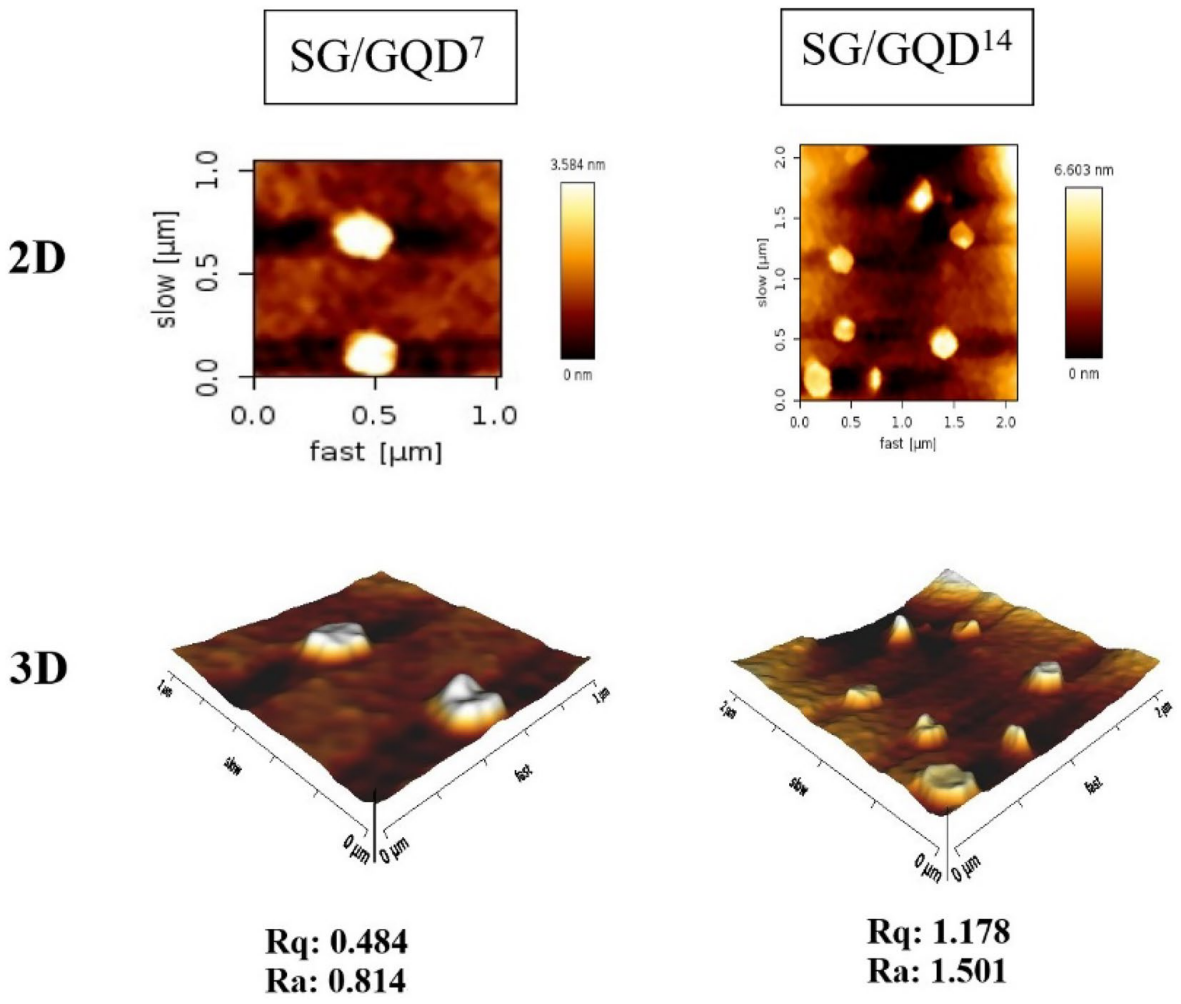
AFM images of nano-biofilms (2D and 3D images).

AFM images in two and three dimensions have been utilized to analyze further the surface topography of packaging nano-biofilms (Fig. 8). Elevated areas on the nano-biofilms appear bright, indicating high height, while indent areas appear dark, denoting low height^57^. Moreover, these contrasting areas serve as GQDs onto the polymer substrate, as the bright dots in the images signify the presence of GQDs in nano dimensions.

The two-dimensional images of the nano-biofilms revealed an uneven surface, with a root mean square (RMS) roughness of 0.814 nm for the nano-biofilm containing 7% quantum dots and 1.501 nm for the nano-biofilm containing 14%. As the amount of GQDs increased, the bright spots grew larger, indicating that the loaded GQDs raised the height of the nano-biofilms. Furthermore, the presence of GQD enhanced the surface roughness of the nano-biofilm layer, leading to an increase in arithmetic average roughness (Ra) from 0.484 to 1.178 and root mean square roughness (Rq) from 0.814 to 1.501.

As observed in the three-dimensional images, the polymer substrate of the nano-biofilms predominantly exhibits a dark portion with a sheet-like surface and moderate surface roughness. When scanning an area of 2 × 2 μm, the SG/GQD^7^ displayed a height of 6 nm, whereas the SG/GQD^14^ exhibited 9 nm.

### An acquaintance with the waterproof quality of nano-biofilms

**Figure 9:**
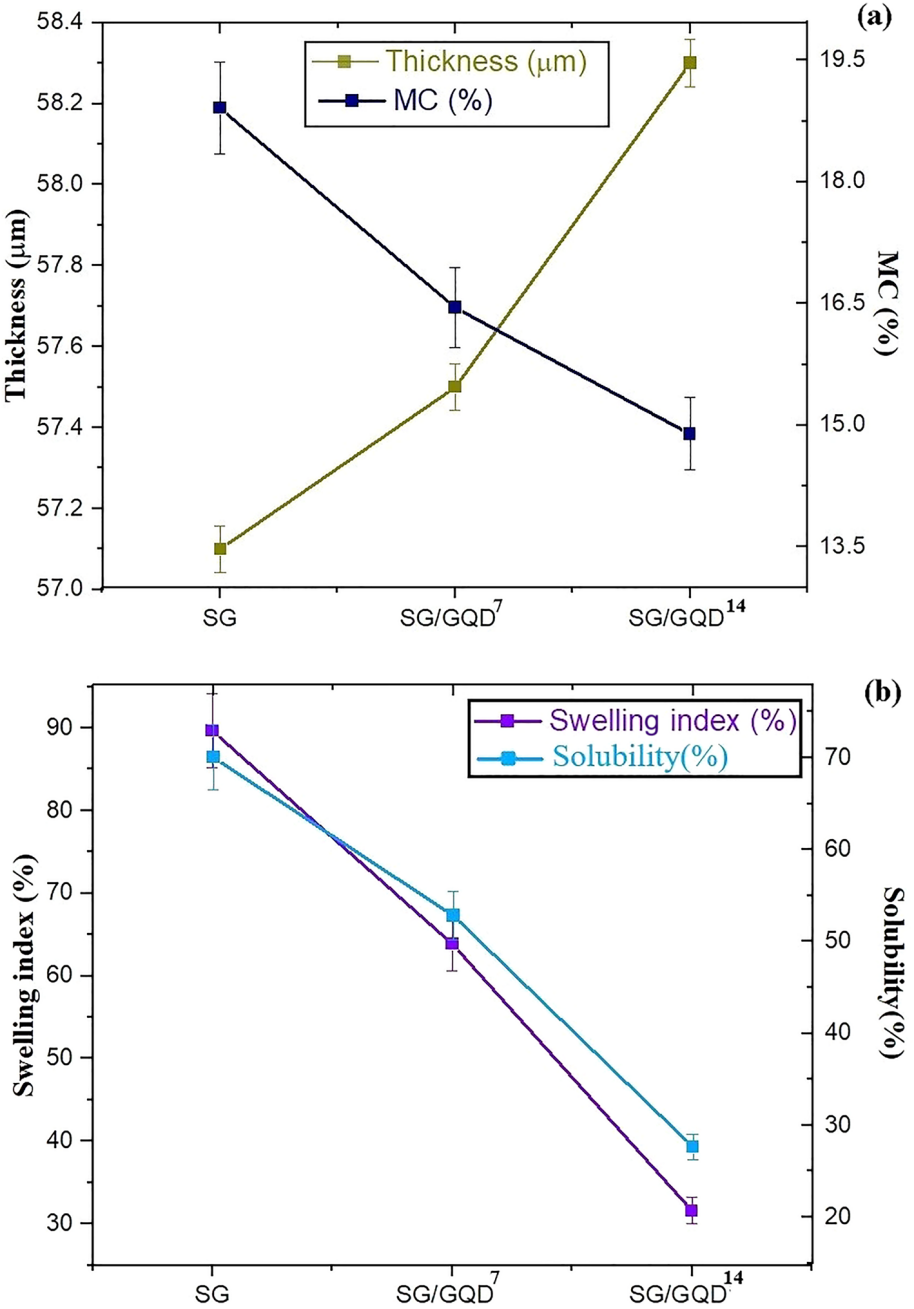
Moisture contact (MC) and thickness (**a**), swelling index (SI), and solubility (S) (**b**) of nano-biofilms by adding GQD.

Packaging films require water resistance as a crucial and fundamental feature. Hence, the films should possess a structure that is not susceptible to moisture caused by food. According to Table 2, the incorporation of GQD into the polymer substrate reduced the moisture contact (MC), swelling index (SI), and solubility (S) by preventing water molecules from penetrating the packaging films through the pores of the matrix (Fig. 9). Moreover, the hydroxyl functional groups present in both GQD and the polymer matrix establish a robust hydrogen bond between them, leading to a reduction in hydroxyl groups in the polymer substrate and a decrease in the hydrophilicity of the films. Furthermore, GQD elevates the twist in the polymer substrate^8,25^.

**Table 2:** Thickness, moisture contact, swelling index, and solubility of nano-biofilms.

Films	Thickness (μm)	Moisture contact (%)	Swelling index (%)	Solubility (%)
SG	57.1	18.91	89.65	70.05
SG/GQD^7^	57.5	16.45	63.87	52.81
SG/GQD^14^	58.3	14.89	31.59	27.63

To create a waterproof layer, it is recommended to utilize packaging materials containing hydrophilic functional groups that can establish hydrogen bonds with food water vapor and interact with dipole–dipole forces. In the case of hydrophilic polymers, it is preferable to incorporate cross-linking agents during the material preparation process to prevent water formation caused by food. This results in the formation of cross-linked polymer networks with a stable structure^58^. To achieve this, in this study two hydrophilic polymers (starch and gelatin) were used, along with GQDs, in addition to glycerin softener.

### Survey optical properties of the nano-biofilms

To appeal to consumers and boost sales, food packaging must have high optical transparency to showcase the freshness and grade of the food^59^. Nonetheless, practicality is also important to conserve the food’s shelf life, so the packaging must have stable protective features against assorted factors. According to Table 3, merging GQD into the substrate can expand the density and viscosity of the films, leading to a decline in transparency. Matte packaging can reflect sunlight and develop a protective obstacle to deter food spoilage.

**Table 3:** Opacity and transparency at 280 and 660 nm.

Films	Opacity (Abs_600_ mm^−1^)	T_660_ (%)	T_280_ (%)
SG	0.96	85.12	51.54
SG/GQD^7^	2.34	76.87	38.70
SG/GQD^14^	5.81	61.56	23.49

**Figure 10:**
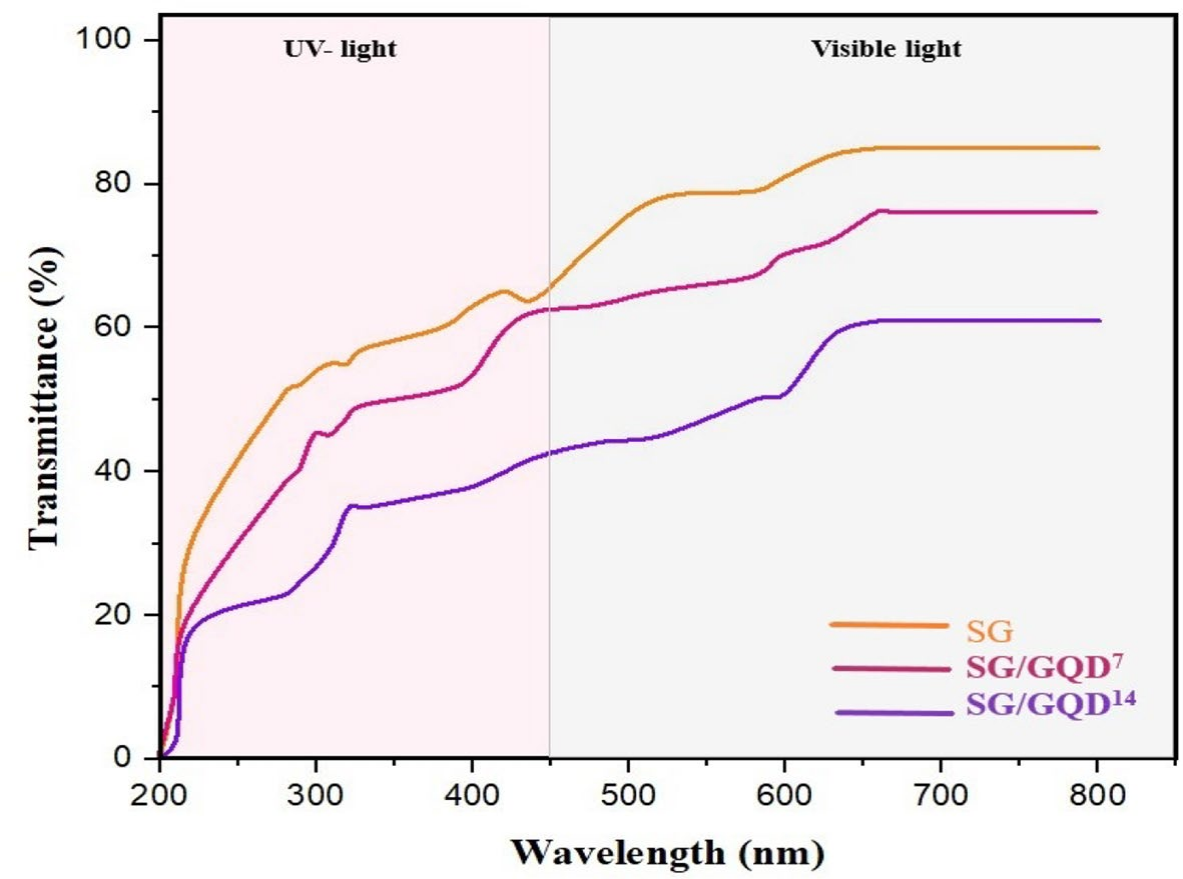
UV–Vis transmittance spectra of starch/gelatin neat biofilm and nano-biofilms containing 7 wt% and 14 wt% of GQD.

Figure 10 displays the light transmittance of the films, revealing a decrease in transmittance in both the visible range (660 nm) and UV region (280 nm) with the incorporation of GQD. This is due to GQD's high light absorption and sensitivity, commonly used to enhance optical sensors^60^. The control film (SG) exhibited greater light transmittance in both the visible and ultraviolet regions due to its exceptional transparency, possibly due to the limited ability of starch and gelatin to obstruct light. While all three types of films displayed high transmittance in the visible region (660 nm), it is crucial to focus on the ultraviolet region, where harmful ultraviolet rays significantly contribute to food spoilage. Among the nano-biofilms, the SG/GQD^14^ film demonstrated superior performance in the UV barrier, with a light transmission rate of 23.49%. This can be attributed to the increased presence of quantum dots in the film, effectively filling a substantial percentage of the film's holes and preventing the passage of light.

### Penetration test of GQDs in food

**Figure 11:**
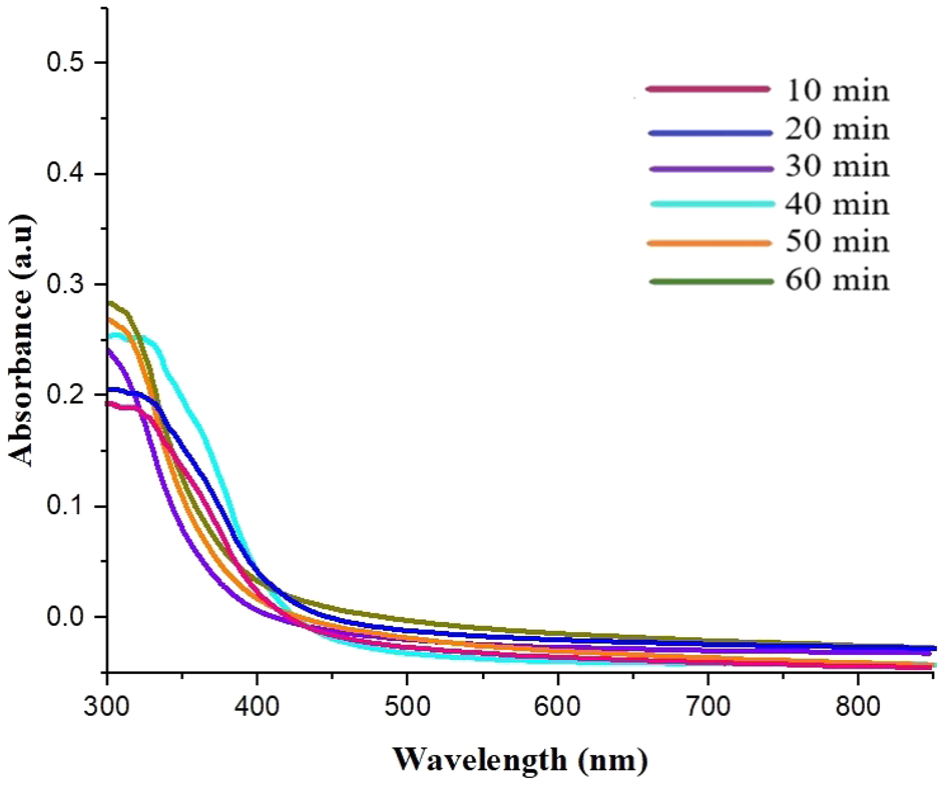
UV absorption diagram of penetration of GQDs into the virtual food model.

To ensure the safety of the synthesized nano-biofilms and prevent contamination of packaged food, the UV absorption calculation of varied film types was utilized in the virtual food model. Figure 11 presents the UV absorption spectrum of the nano-biofilms at time intervals ranging from 10 to 60 min. All spectra exhibited a peak at 340–360 nm, indicating the presence of GQDs. This information suggests that the nano-biofilms, which were coated with GQDs were able to maintain their stability and prevent contamination of the packaged food. The UV absorption calculated showed that the films had a consistent absorption rate below 0.3, indicating that the GQDs had minimal penetration into the virtual food model. This suggests that the GQDs remained stable within the polymer matrix of the nano-biofilms and did not leach into the food. Overall, these findings support the safety and effectiveness of using GQD-coated nan0-biofilms for packaging food.

### The antioxidant performance of nano-biofilms

The method is founded on the reduction of DPPH free radicals by antioxidants in the scarcity of other free radicals, authorizing the inspection of antioxidant power. DPPH, a stable free radical with an unpaired electron on one of the nitrogen bridge atoms, functions as an electron acceptor from a donor molecule such as an antioxidant, converting DPPH to DPPH_2_ and altering the color of the environment from violet to yellow. The decrease in absorption intensity at 515 nm can be measured by spectrometry to determine antioxidant properties^61^.

**Figure 12:**
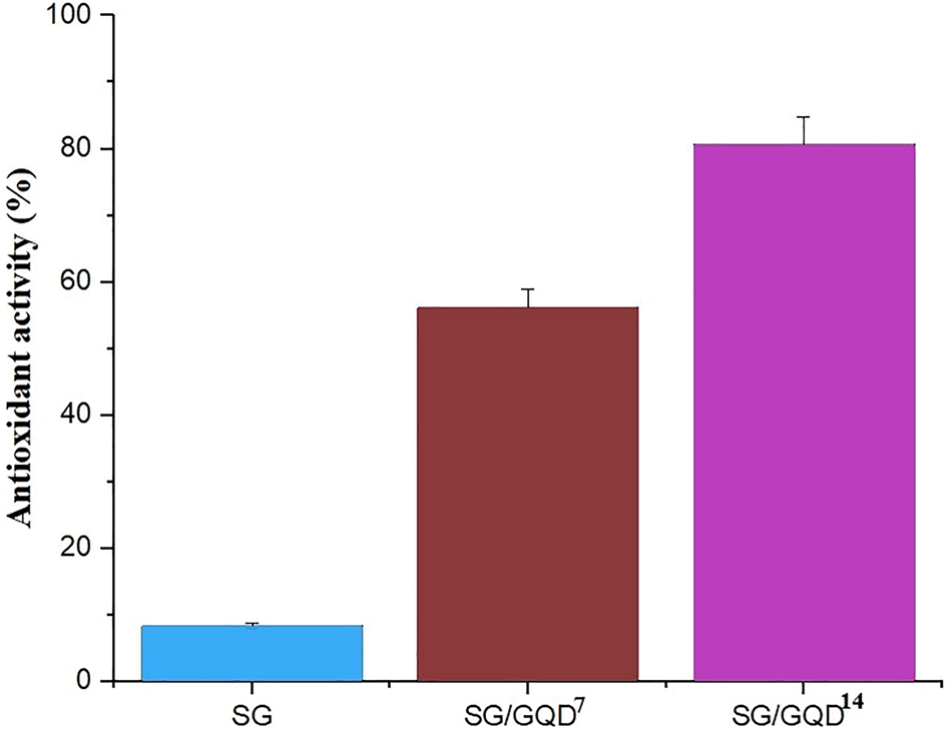
Antioxidant activity of starch/gelatin neat biofilm and nano-biofilms containing 7 wt% and 14 wt% of GQD.

During the warehouse of packaged foods, oxidation not only reduces quality but also lessens nutrients^62^. Antioxidants are used to deter this process. GQDs have OH groups that function as electron donors that are accepted by DPPH free radicals and quench them. These belongings of quantum dots help to prevent further free radicals′ reactions and safeguard food from oxidation. Figure 12 illustrates the effectiveness of this cycle, where the control film (SG) showed only 8.34% for the DPPH test. In comparison the SG/GQD^7^ demonstrated 56.14% and 80.76% for SG/GQD^14^, indicating the high antioxidant capacity of GQD.

### Investigating the antimicrobial activity of the films by disk diffusion method

**Figure 13:**
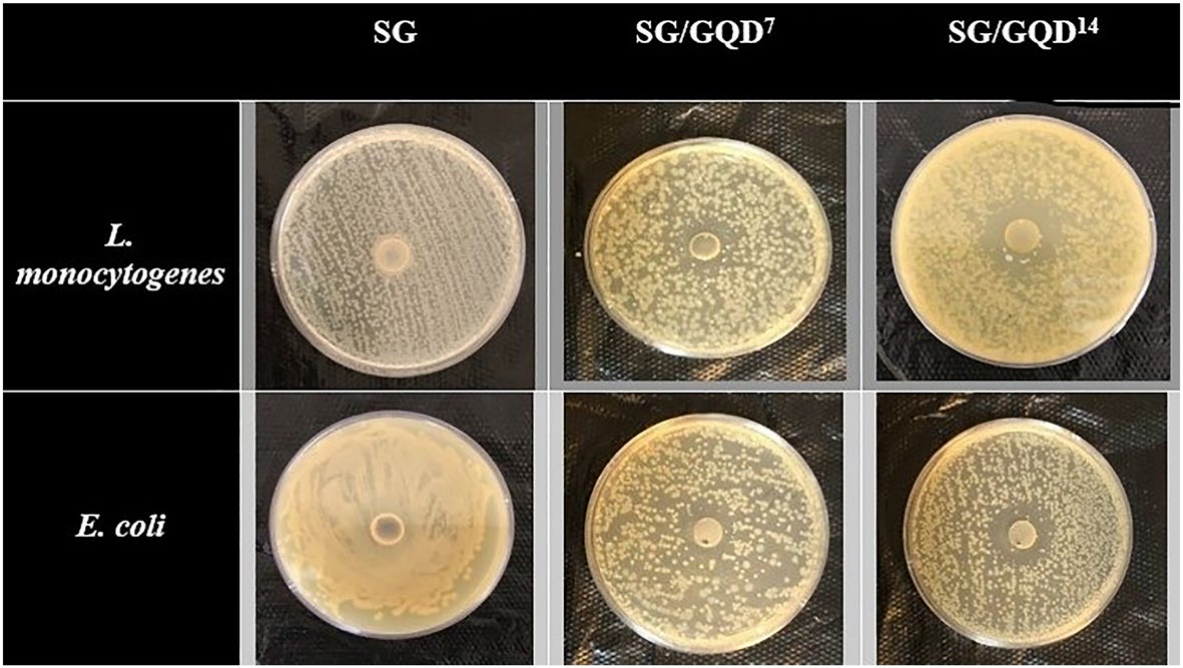
The zone of inhibition (ZOI) images and diameters of starch/gelatin neat biofilm and nano-biofilms containing 7 wt% and 14 wt% of GQD.

Antimicrobial packaging (AMP) is a specialized form of active food packaging that utilizes varying amounts of antibacterial implications to keep the shelf life of food and prevent spoilage. The proliferation of GQD to the polymer substrates in this study also qualifies as AMP, as it adheres to the four fundamental tenets of including a polymer, solvent, antibacterial meanings, and secure agents. Based on the classification system for antibacterial agent's cogency in bacteria destruction, which is defined by the diameter of the inhibition zone, films comprising GQD fall under the fourth criterion denoting extreme sensitivity, demonstrating GQD's exceptional proficiency to eradicate bacteria^63^. Figure 13 displays the zone of inhibition (ZOI) size in the nano-biofilms. The control film (SG) showed no ZOI, but the SG/GQD^7^ film had 22 mm against *L. monocytogenes* and 20 mm against *E. coli*. The results of the SG/GQD^14^ film were much better than the previous two films, as it had a ZOI of 28 mm versus *L. monocytogenes* and 25 mm for *E. coli*.

The films (SG/GQD^7^ and SG/GQD^14^) show greater effectiveness against gram-positive bacteria (*L. monocytogenes*) as compared to gram-negative ones (*E. coli*). This may be because gram-positive bacteria have a more permeable cell wall owing to its consistency, and GQD can penetrate it. Upon penetration, it targets the genetic structures of the bacteria, directing their disruption, collapse, and destruction^47^.

### Decomposability of nano-biofilms

**Figure 14:**
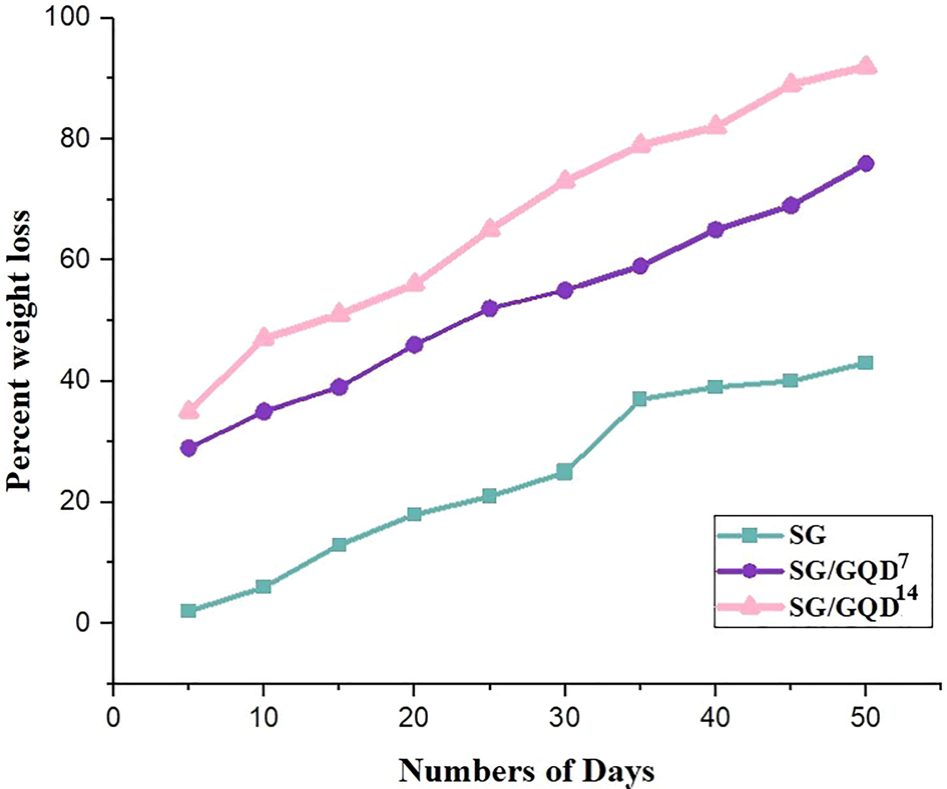
Decomposability of starch/gelatin neat biofilm and nano-biofilms containing 7 wt% and 14 wt% of GQD.

The decomposition of the synthesized nano-biofilms was investigated over 50 days using the soil burial method. The results, shown in Fig. 14, demonstrate that film degradation primarily occurs through hydrolysis. The presence of water is crucial for the degradation process, as it enables the growth and proliferation of microorganisms that contribute to the breakdown of the polymer layer. Additionally, the involvement of water molecules in the hydrolysis process results in the infringement of H-bonds within the polymer chains, causing the formation of slighter pieces. The degradation procedure is further accelerated by the hydrophilic properties of the nano-biofilm components^58^.

The degradation percentage in each nano-biofilm is influenced by the composition and nature of the components. Moreover, nano-biofilms containing GQDs exhibit greater degradation efficiency compared to polymer nano-biofilms. Based on the obtained results, the fabricated nano-biofilms have the potential to serve as a viable alternative to synthetic and environmentally friendly packaging films.

### TS, EAB, and YM of nano-biofilms

The food packaging industry relies heavily on materials with strong mechanical properties to ensure that packaging can withstand the rigors of storage and transportation. For instance, the TS of a film reflects its ability to resist stress, while the EAB measures its ability to stretch without breaking. Additionally, the YM indicates the rigidity of the film. These properties are crucial for ensuring that food packaging can maintain its integrity and protect the contents throughout the supply chain. Therefore, improving mechanical properties is a key focus for the food packaging industry to enhance product quality and safety^39^. The article confirms that GQDs and polymer matrix have high compatibility, which has been verified through various tests such as FT-IR, XRD, and FE-SEM. The mechanical properties of the films are dependent on the interaction between the filler nanoparticles and the polymer substrate. By adding GQD to the polymer matrix, the mechanical properties of the films have been improved. Table 4 shows that although the flexibility of the nano-biofilms decreases with an increase in nanoparticles, the strength and stiffness of the films increase. This improvement in mechanical properties can better protect packaged foods.

**Table 4:** Tensile strength (TS), elongation at break (EAB), and Young’s modulus (YM) of nano-biofilms.

Films	TS (MPa)	EAB (%)	YM (MPa)
SG	49.21	11.92	760.20
SG/GQD^7^	71.05	9.13	1354.53
SG/GQD^14^	83.19	8.65	3009.24

### Application of nano-biofilms enhancing shelf-life of whole cherry and cucumber slices

**Figure 15:**
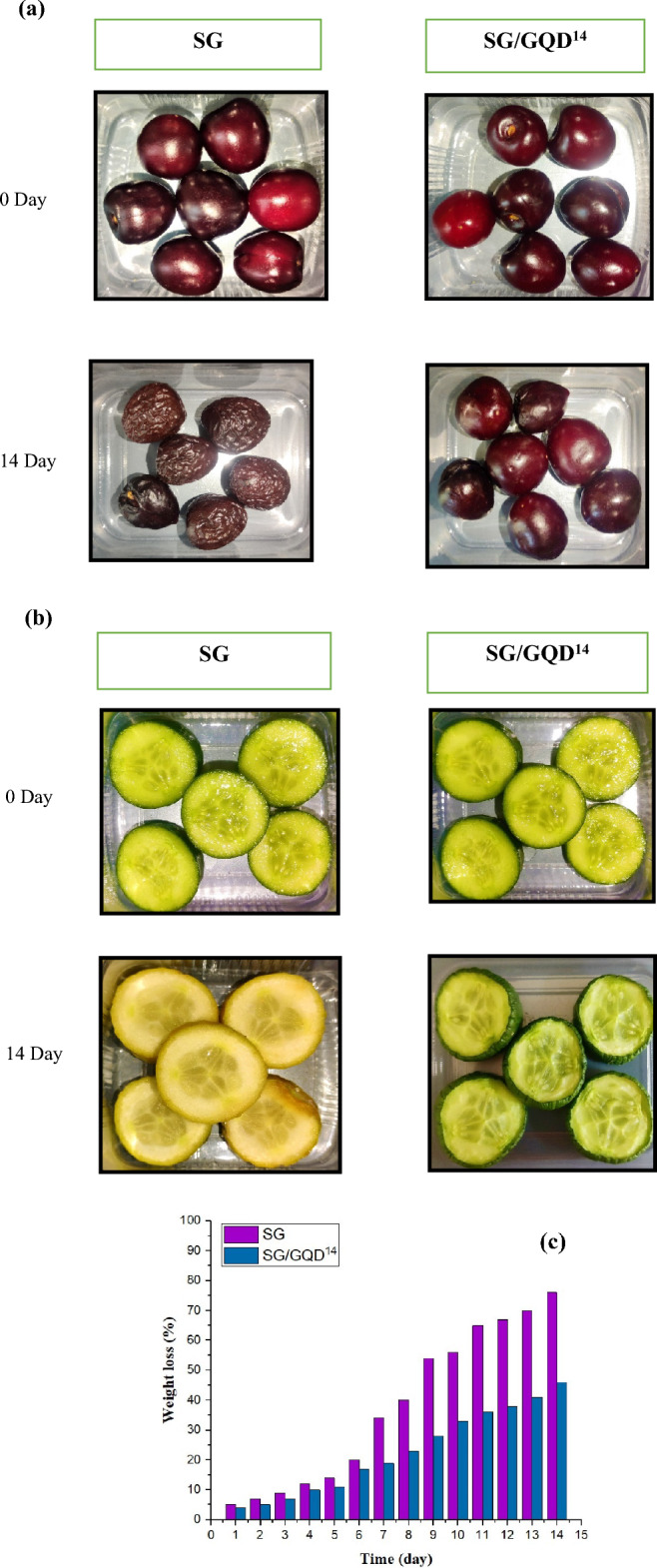

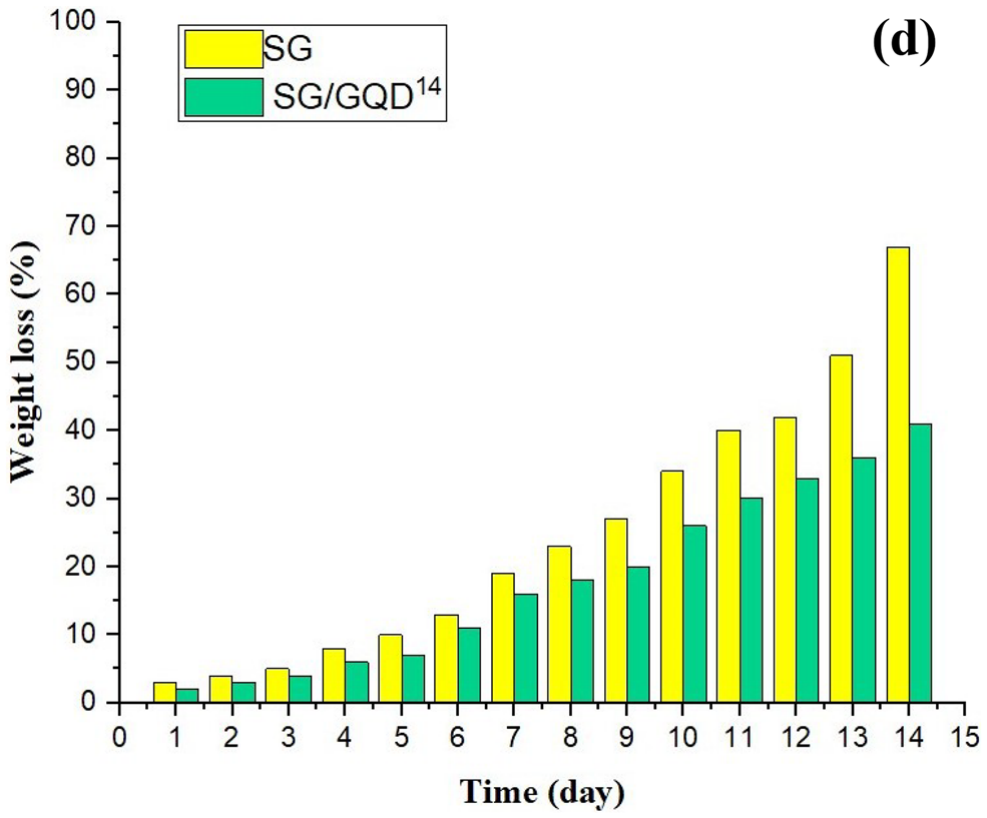
Protection test of cherries (**a**) and cucumbers weight losses of packaged fruits (**b**) (cherries (**c**) and cucumbers with and without GQDs (**d**).

Coatings containing GQD were utilized as a protective agent for the preservation of fruits and vegetables due to their potent antioxidant capacity and antimicrobial properties. As depicted in Fig. 15a, the cherries exhibited a fresh and juicy impression at the start of the experiment. However, after 14 days of storage in the ambient air, the cherries wrapped with the SG control film appeared severely shriveled and had undergone significant water loss, whereas the fruits covered with the SG/GQD^14^ nano-biofilm retained their freshness.

After 2 weeks, cucumbers covered with the control film turned yellow and showed signs of microbial spoilage, whereas cucumbers covered with GQDs remained unaffected. The nano-biofilms with GQD significantly improved the quality of cucumbers and effectively prevented microbial contamination during storage (Fig. 15b). A coating of SG/GQD^14^ proved to be effective in preventing tissue damage, browning, and nutrient loss, thus extending their shelf-life^64^.

Additionally, when food loses water, it can also lead to a loss of nutrients. Water-soluble vitamins and minerals can leach out of the food, resulting in a decrease in nutritional value. Dry and less appealing food can be unpalatable and unenjoyable to eat. By preserving moisture, the food can maintain its desired texture and taste, making it more appetizing and satisfying. The amount of water lost from the fruits was determined by measuring the decrease in their weight, and the findings are presented in Fig. 15c,d. The weight loss of all fruits slowly boosted with time, but this transition was not enormous in fruits covered with GQDs and they still retain their quality.

## Conclusions

The synthesis of GQDs via citric acid pyrolysis is a widely used method that allows for the precise control of the size and properties of the GQDs. The crystallinity size of the GQDs was determined to be 37.1 nm using Scherer's equation. Nano-biofilms were created by incorporating 7 and 14 wt% of GQD (based on polymers) into a matrix consisting of natural polymers such as starch and gelatin. The resemblance between the FT-IR and XRD spectra of all kinds of packaging films can be attributed to the strong fusion and interaction between the GQD and the polymer matrix. The GQDs were effectively embedded within the matrix, forming an obstacle against spoilage agents, as evident in the FE-SEM images. AFM analysis showed that adding GQDs to the nano-biofilms matrix increases the roughness on the film surface, so the RMS increased from 0.814 nm (SG/GQD^7^) to 1.501 nm (SG/GQD^14^). The antibacterial activity against both Gram-positive and Gram-negative bacteria (*L. monocytogenes* and *E. coli*) was illustrated to have a ZOI of more than 20 mm, which can be attributed to the strong bactericidal properties of GQDs. Additionally, the films showed good compostability and effectively increased the shelf life of cherries and cucumbers by preventing spoilage and water loss. These results highlight the potential of GQDs as safe and promising nanoparticles for food preservation, addressing concerns about the environmental accumulation of synthetic plastics and associated health risks.

## Data Availability

All data have been given in the manuscript.
